# Effects of monomer contamination during post-rinsing in vat photopolymerization on dimensional stability

**DOI:** 10.1038/s41598-025-94404-4

**Published:** 2025-03-24

**Authors:** Florian Flierl, Benedikt C. Spies, Severin Rothlauf, Kirstin Vach, Ralf J. Kohal, Jörg Lüchtenborg

**Affiliations:** 1 Center for Dental Medicine, Department of Prosthetic Dentistry, Faculty of Medicine, University of Freiburg, Hugstetter Str. 55, 79106 Freiburg, Germany; 2 Institute of Medical Biometry and Statistics, Faculty of Medicine, University of Freiburg, Stefan-Meier Str. 26, 79104 Freiburg, Germany

**Keywords:** Accuracy, Post-rinsing, Monomer contamination, Standardized test specimen, Surface roughness, Engineering, Aerospace engineering, Biomedical engineering, Mechanical engineering

## Abstract

Accuracy is an important parameter of additively manufactured objects. The influence of post-processing on the objects, in particular the effects of monomer contamination in isopropanol used during the post-rinsing of parts produced by vat photopolymerisation, is therefore important. Forty test specimens were fabricated from standard dental resin and divided into four groups, each rinsed with isopropanol containing 0 wt%, 10 wt%, 20 wt% or 30 wt% resin contamination. Optical scans of the specimens were compared with the original design to assess dimensional deviations. The results showed no significant deviations at contamination levels up to 10 wt%. At 20 wt%, deviations were observed in small structures and inclined planes, while 30 wt% contamination resulted in significant deviations in all areas. Rinsing with 99.9% isopropanol consistently produced the highest accuracy. Resin build-up below 10 wt% did not affect dimensional stability, suggesting that isopropanol replacement is unnecessary at this level. However, a contamination level of 20 wt% can lead to deviations under certain conditions, and a contamination level of 30 wt% consistently affects accuracy. These findings highlight the importance of monitoring contamination levels to maintain the integrity of vat photopolymerisation workflows and suggest that isopropanol should be replaced at contamination levels approaching 30 wt%.

## Introduction

Additive manufacturing (AM) is a widely adopted technology in various industries, particularly valued for rapid prototyping and the production of complex structures. Various AM technologies, each with unique advantages, are utilized across different fields. One commonly used method is vat photopolymerization (VPP), which involves curing layers of liquid photopolymer resin using a light source. The VPP process includes several key stages: pre-processing, processing, and post-processing. During pre-processing, the digital design is prepared, sliced in printable layers and a support structure is added to the object. The actual processing phase involves the layer-by-layer construction of the object, where the resin is selectively cured to form the desired shape. Finally, post-processing involves steps such as removing support structures, cleaning uncured resin, and further curing the object. In this often underestimated phase of the AM process, the final properties like the mechanical stability but also the degree of accuracy of the object are obtained. A vital aspect of post-processing is the washing process, which typically uses solvents like isopropanol (IPA) to remove uncured resin, consisting of the different monomers, from printed parts, which accumulates then in the rinsing solution. The effectiveness of this washing process can be influenced by the condition of the solvent, particularly its resin concentration. There are already numerous publications on assessing the accuracy of additively manufactured components and parameters such as the duration of the washing process [1–3], type of solvent [2] or the effects of manual and automated post-rinsing [1, 4] have already been investigated. However, the effect on the accuracy of VPP-workflow of an increasing monomer/resin contamination in the IPA bath is the subject of this work. The accumulation of monomers occurs during the continuous use of solvent baths and increases with the number of post-rinsed printed parts. This parameter, which has so far been poorly researched, is therefore of enormous importance in daily use, especially if replacement is not carried out for reasons of cost or negligence, for example.

The choice of the test specimen has an impact on the transferability of the results. Some studies focused on general, basic geometric shapes as a test specimen to investigate accuracy and dimensional stability, including geometric structures such as bars [2, 5–7] and discs [5, 8]. The use of these structures provides a broad understanding of accuracy and dimensional stability when comparing mesh surfaces and computer-aided design (CAD) models. However, these simple test specimens fail to represent the complex and highly individualized shapes of numerous 3D-printing applications that are used in a diverse range of areas. This limitation makes it challenging to generalize the results obtained from these studies to actual, practical application. For this reason, various specific test specimens were examined, depending on the area of use.

In the engineering sector, for example, aircraft turbine blades have been examined for their accuracy [9], which provides more relevant information than test specimens such as bars or discs. In the domain of (bio-) medical research, ceramic or polybutylene succinate scaffolds, which are used as bone grafts or for the cultivation of human mesenchymal stem cells, were examined for surface roughness and porosity [10, 11]. In addition, PEEK disk samples were investigated for the use in orthopaedic applications [12]. Pinto et al. examined models of cadaveric phalanges, that were additively manufactured on the basis of computer tomography, for deviations [13]. Tomaževič et al. [14] investigated AM surgical guides and fixation splints. The corresponding operation to treat a fracture was performed on a model and the different dimensions of the fracture gap were then measured [14]. In the field of dental technology and dentistry, researchers have investigated dental-specific test specimens, including schematic and exemplary dental crowns [15–24], dental bridges [25, 26], surgical guides [4, 27], inlays [28], denture bases, denture teeth and complete dentures [1, 29–32] as well as bite splints [33]. The use of these dental-specific test specimens provides a more practical and application-oriented approach. However, due to the individualized nature of dental prostheses and anatomical variations, analyzing one single exemplary test specimen can only provide limited insights into the properties of all similar specimens.

It is therefore challenging to apply these findings to real-world scenarios. In order to obtain generally valid and transferable results, it is essential to identify a test specimen that contains standardized schematic structures of different sizes and shapes resembling practical applications. Minetola et al. presented a test geometry that is used to investigate convex and concave curved planes [34]. Malesh et al. developed a test artifact, which represents ramps, cones, hemi-spheres, and overhanging features [35]. Moylan et al. introduced a test geometry specifically designed to meet the requirements of AM [36], which served as the basis for a sample for this study.

When examining the accuracy or inaccuracy of test specimens in previous research, certain measurement variables are of particular interest. These variables can be categorized into three-dimensional measurements, such as surface deviations based on mean values [4, 18, 19, 23, 29–31, 33, 37], root mean square RMS or root mean square error RMSE [1, 16, 17, 22, 25, 28, 32] and surface roughness [5, 8–12, 26]. The two-dimensional profile comparisons assess distances between measurement points on defined profile lines [15, 20, 21, 27]. The measurements are based on a comparison of scanned data and the original digital design. In order to ensure detailed results, all the aforementioned measurement variables were considered. The specification of surface deviation as root mean square (RMS) enables the identification of both positive and negative discrepancies. It is important to note that even with a low mean deviation, one single significant discrepancy can result in poor fit of the AM-component. Therefore, an analysis of maximum deviation was incorporated, which can be effectively combined with RMS deviation in three-dimensional and profile-based comparisons. Surface roughness Sa is also a pertinent factor in AM. Printing resolution in the z-direction often leads to visible steps on surfaces that are not parallel to the x–y plane. This phenomenon is frequently observed in organic, anatomically shaped components.

The following null hypothesis H_0_ was assumed: Increasing monomer contamination of the isopropanol solution has no significant effect on the accuracy (mean, max., RMS-deviation; surface roughness) of components that have completed post-processing.

## Methods

### Test specimen and measurement parameters

**Fig. 1:**
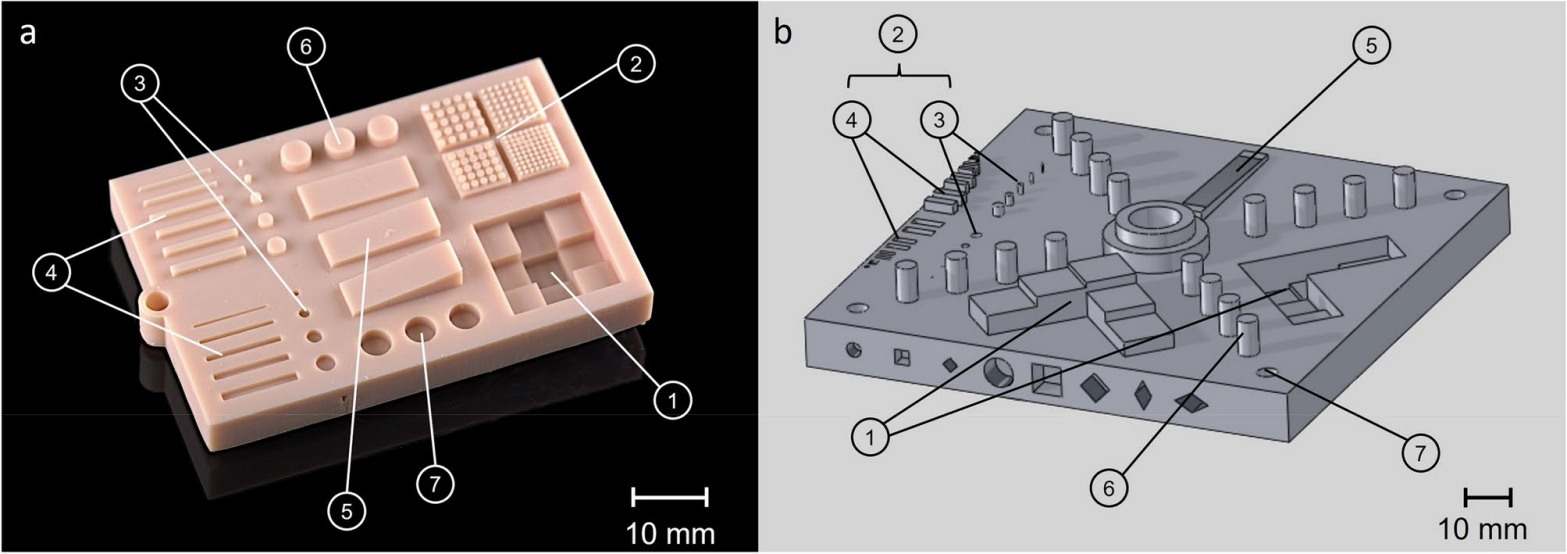
(**a**) The test specimen is based on a (**b**) standardized test specimen that was presented by Moylan et al. [36]. The measurement structures are: (**1**) Staircase-Structure; (**2**) Set of fine features; (**3**) Small pins / small holes; (**4**) Cuboid bars / cuboid cavities; (**5**) Ramps; (**6**) Medium pins; (**7**) Medium holes.

A test specimen has been developed that allows to evaluate the dimensional stability of a wider range of different geometries (Fig. 1a). The test specimen was based on a standardized test geometry, developed by Moylan et al. (National Institute of Standards and Technology, NIST, USA), which has been proposed for the purpose of evaluating the performance of different additive manufacturing systems [36]. The specimen is shown in Fig. 1b. It had an edge length of 140 mm and a volume of 101,000 mm^3^. This standardized, relatively large test specimen was modified according to the following criteria [38], so that it could be used in the present study to evaluate the accuracy of the VPP workflow of a rather small “desktop” printer:

- The specimen should be large enough to evaluate performance near the edges and center of the geometry base.
- It should include a wide range of small, medium, and large dimensions.
- The specimen should include features that resemble “real parts”.
- The dimensions should include both “inside” and “outside” measurements.
- The specimen should be suitable for measurement purposes.
- No support structure is necessary, so it can be placed directly on the building platform.
- Its dimensions allow testing of industrial systems as well as low-cost “desktop” printers with small construction space.
- There are no overhanging features, which would be more difficult to capture by optical scanning methods.

The resulting specimen consists of a body with dimensions 60 × 40x9 mm and several artefacts (STL data available in the supplementary material). The dimensions of the sample and the unused surface area of the base have been minimized and the number of redundant structures reduced. The lateral structures were excluded due to the difficulties encountered in the optical detection process. The sample body contained no undercuts and could be produced without a support structure. A suspension eyelet was provided for carrying out post-rinsing in a beaker.

The specimen of the present investigation (Fig. 1a) contained the following measurement structures, which were analyzed for the parameters described in paragraph 2.3:

#### Staircase-structure

Quadratic stages (5 × 5 mm) were arranged in three levels of depth to top surface (-2; -4; -6 mm) in order to visualize maximum deviation from the CAD-file by creating a 3D-heatmap through Geomagic Control X (3D-Systems, Rock Hill, USA).

#### Set of fine features

Bars with a height of 0.5 mm were arranged in four fields (7 × 7 mm). Diameter (0.5 mm; 1 mm) and distance (0.25 mm; 0.5 mm) are increasing. The analysis was performed with Geomagic Control X. Based on this structure, the deviation from the original CAD-file was measured and presented as Root Mean Square (RMS) and maximum deviation.

#### Small pins and holes

The pins and holes had a height/depth of 1 mm and increasing diameters (0.5; 1; 1.5; 2; 2.5 mm). They were arranged with their center point in one line. Analysis was performed using Keyence VR-5000 software (Keyence Corporation, Osaka, Japan). A virtual section plane was placed through the central axis of the cylinders. In the area of the cylinders, the average and maximum deviation along the entire length of the two profile lines were determined.

#### Ramps with increasing inclination

The three ramps equal in size of 5 × 15 mm were designed to rise constantly with a defined inclination (0°; 5°; 10°) to measure the surface roughness, which depends on resolution in the z-axis. The layer thickness caused a visible “stair stepping” effect. Surface roughness was measured at defined areas with a distance of 0.5 mm to the edge. If the measuring range is extended directly to the edge, there is a risk of measuring errors, since in some cases the distance to the base surface can be detected, resulting in falsely increased measured values. The evaluation was carried out with Keyence VR-5000 software.

### Additive manufacturing and post-processing

A total number of 40 test specimens were produced, using a Formlabs Form 3 printer and Model V2 resin (Formlabs Inc., Somerville, USA). Model V2 resin is Formlabs’ proprietary resin for producing high-precision dental models and is widely used in dental laboratories. The printing resolution in the z-axis was 25 µm. These specimens were divided into four groups of 10 samples each. Subsequently, the specimens from each group underwent a washing process in IPA of varying purity levels to simulate different levels of monomer contamination. The IPA used initially had a purity of 99.9% (Höfer Chemie, Kleinblittersdorf, Germany). Model resin was then added to achieve purities of 90%, 80%, and 70%, resulting in monomer contaminations of 0 wt%, 10 wt%, 20 wt%, and 30 wt% respectively. 0 wt% reflects fresh IPA immediately after it is replaced. 10 wt% is the upper limit commonly statet by the manufacturer. 20 wt% and 30 wt% were chosen to represent real-world scenarios where contamination exceeds recommended thresholds. The washing procedure was conducted in a beaker for 10 min using a magnetic cylindrical stirrer with a stirring speed of 500 rpm and a stir bar length of 15 mm. Following washing, the samples were cured following the manufacturer recommondations in a Form Cure at 60 °C for 60 min.

### Sample digitalization, measurement structures and accuracy tests

To assess accuracy, the test specimens were scanned using a 3D-profilometer (Keyence VR-5000, Keyence Corporation, Osaka, Japan) with a measurement accuracy of ± 4 µm for the height and ± 5 µm for the width and a reproducability of 1 µm. The system was calibrated using the ceramic calibration standard block OP-88275, which has an uncertainty of ± 2 μm. The resulting data was converted into Standard Tessellation Language (.stl) files. The scan protocol was created using a sample from the 0 wt% contamination group. A specific area of the sample was defined (staircase-structure with the outer edge of the sample body) that could be unmistakably recognized by the Keyence software when scanning the other samples. The following samples and their individual measurement structures were aligned accordingly. Accuracy was then analyzed, Keyence VR software was employed to assess profile deviation and surface roughness, while Geomagic Control X software, version 2022.0.0. (3D-Systems, Rock Hill, USA) was used to evaluate RMS and maximum 3D-surface deviation. For this purpose, in Geomagic Control X software the original CAD-file was aligned with the scanned specimen surface using an initial fit (manual alignment using three specific points on the surface), followed by a Gaussian local best-fit algorithm for precise superimposition of the sample surface with original CAD.

### Statistics

With a sample size of 10 in each group and based on previous studies on accuracy [1, 16–18, 31, 32], an effect size of 1.3 could be demonstrated with a 5% error probability and a power of 80%. Mean values and standard deviations were calculated for descriptive analysis. Since the data did not follow a normal distribution, non-parametric methods were applied. The Kruskal–Wallis test was used to assess group differences across the various outcome variables. For subsequent pairwise comparisons, Dunn’s test was employed, with corrections for multiple testing using the Holm method. The statistical analysis employed three different significance thresholds: 0.05 (low significance), 0.01 (medium significance), and 0.001 (high significance). Observations with *p* > 0.05 show no significance. All analyses were conducted using STATA (Version 17.0, College Station, TX, USA).

## Results

**Fig. 2:**
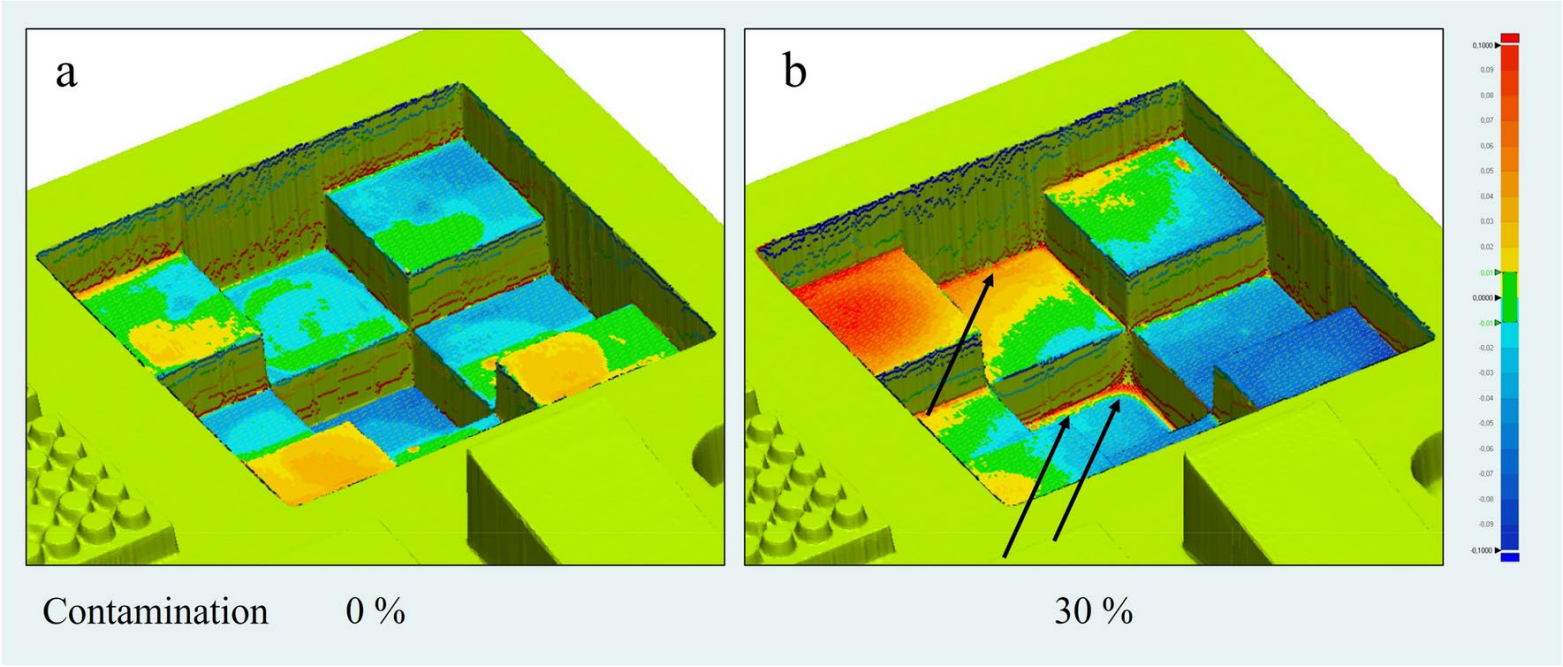
Representative sample of “Staircase” structures washed (**a**) non-contaminated and (**b**) 30 wt% contaminated IPA. Resin accumulation can be observed mainly in the edges and corners (black arrows). The figures were taken from the evaluation by Geomagic Control X software, version 2022.0.0. (https://oqton.com/geomagic-controlx/).

**Fig. 3:**
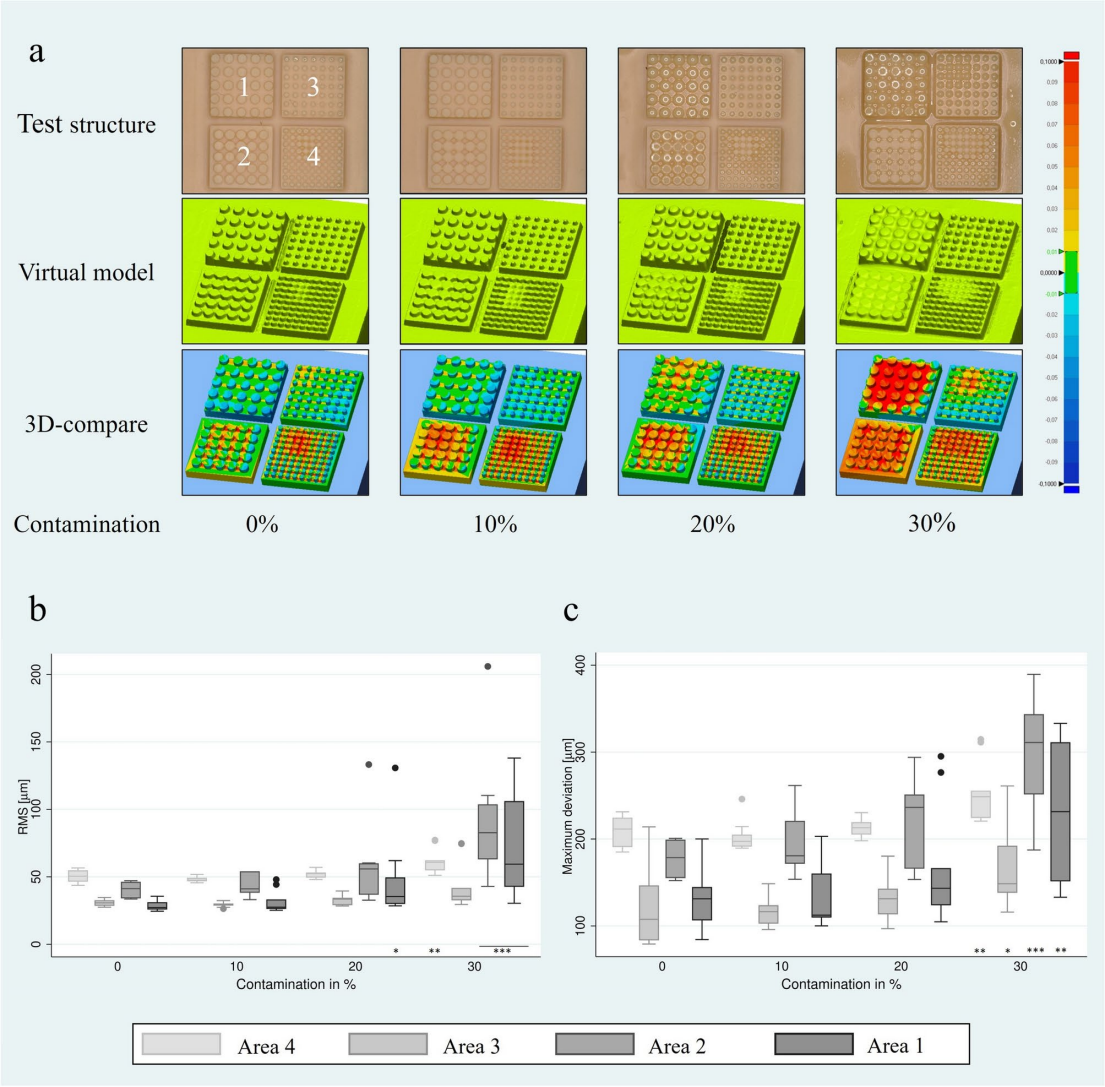
Deviation of the samples from the CAD-file after the washing process: (**a**) Overview of representative samples containing 4 test areas. The figures were taken from the evaluation by Geomagic Control X software, version 2022.0.0. (https://oqton.com/geomagic-controlx/). Deviation of the 4 tested areas in (**b**) root mean square RMS and (**c**) maximum deviation with relation to differences in distance and diameter of columns and the contamination concentration of isopropanol. The highest deviation was visible for samples with contamination of 30 wt%. Significance against post-rinsing with non-contaminated IPA: *p < 0.05, **p < 0.01, ***p < 0.001.

A particularly large amount of resin accumulation occurred in the corners and edges from a contamination level of 20 wt% (see Fig. 2a-b, contamination 30 wt%). The results of the surface deviation in RMS and maximum deviation are shown in Fig. 3a-c. Significant deviations were observed in all areas particularly in the 20 wt% and 30 wt% contamination group. In addition, the resin accumulation is shown in comparison of the test specimen to the virtual scan model. The heatmap shows deviations from + 100 µm (red) to -100 µm (blue), with deviations of ± 10 µm marked in green.

**Fig. 4:**
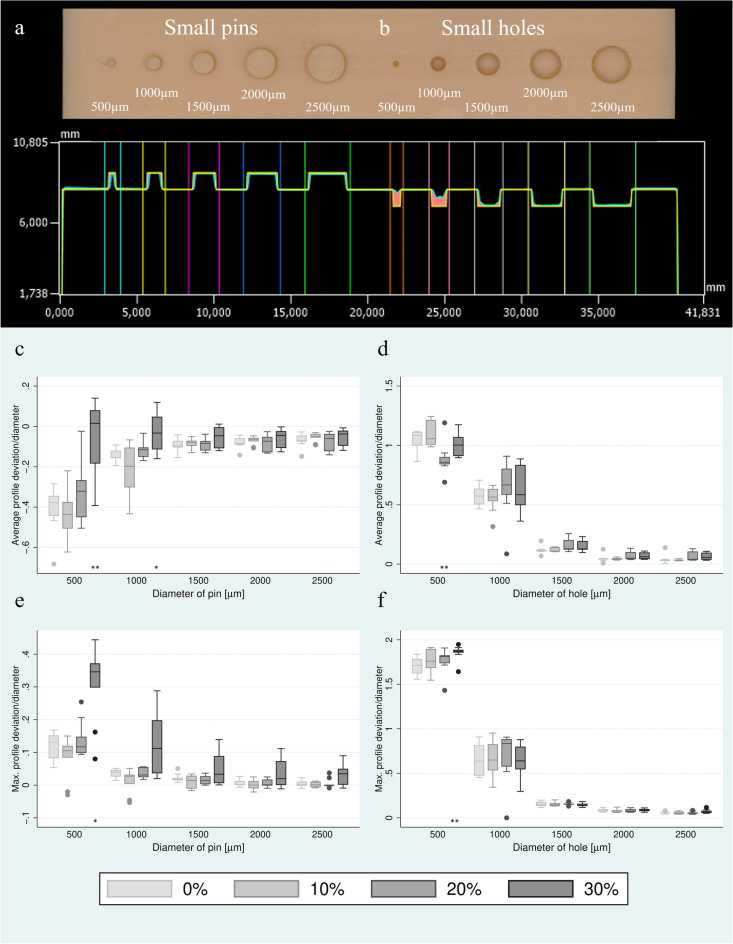
Overview and cross section of exemplary (**a**) small pins and (**b**) small holes structure. The cross section figure was taken from Keyence VR-5000 software (https://www.keyence.de). Average profile deviation of (**c**) pins and (**d**) holes. Maximum profile deviation of (**e**) pins and (**f**) holes. All values given are normalised to the diameter of the test specimen. Significance against post-rinsing with non-contaminated IPA: *p < 0.05, **p < 0.01, ***p < 0.001.

The average and maximum deviation from the CAD-file in the cross-section through small pins and holes are shown in Fig. 4a-f and Table 1. Positive deviations are marked in red and negative deviations in blue. The measured values are normalised to the diameter of the respective structure. Significant deviations occurred only in the area of very small structures: small pins with a diameter of 500 µm (mean and maximum deviation) and 1000 µm (mean deviation) and small holes with a diameter of 500 µm (maximum deviation) showed significant deviations when washing in the 30 wt% contamination group. Likewise, these occurred also when washing in 20 wt% contamination group at small hole structure with a diameter of 500 µm.

**Table 1:** Small pin and hole structures, average profile deviation in absolute values [in µm].

	Pin and Hole Diameter	500 µm	1000 µm	1500 µm	2000 µm	2000 µm
Small Pin Structure	0 wt% Contamination	− 203.4 ± 54.1	− 145.7 ± 29.6	− 139.4 ± 43.2	− 161.4 ± 52.7	− 171.8 ± 78.8
	10 wt% Contamination	− 212.6 ± 57.3	− 216.1 ± 115.2	-126.7 ± 37.9	− 140.7 ± 38.7	− 135.7 ± 49.5
	20 wt% Contamination	− 155.7 ± 76.7	− 116.3 ± 37.0	− 130.5 ± 45.9	− 166.2 ± 80.7	− 194.2 ± 112.8
	30 wt% Contamination	− 32.2 ± 88.9**	− 30.0 ± 92.7*	− 77.7 ± 72.7	− 115.9 ± 89.1	− 127.4 ± 96.7
Small Hole Structure	0 wt% Contamination	523.8 ± 39.0	577.8 ± 82.6	177.1 ± 44.3	92.9 ± 57.1	100.3 ± 84.6
	10 wt% Contamination	544.5 ± 46.8	552.0 ± 98.7	180.5 ± 26.2	88.5 ± 16.1	87.0 ± 13.1
	20 wt% Contamination	441.0 ± 59.7**	642.3 ± 224.6	240.8 ± 79.5	145.9 ± 73.4	164.6 ± 95.7
	30 wt% Contamination	502.3 ± 44.6	637.8 ± 191.6	232.3 ± 72.5	133.8 ± 51.7	160.5 ± 71.4

Significance against post-rinsing with non-contaminated IPA: *p < 0.05, **p < 0.01, ***p < 0.001.

**Fig. 5:**
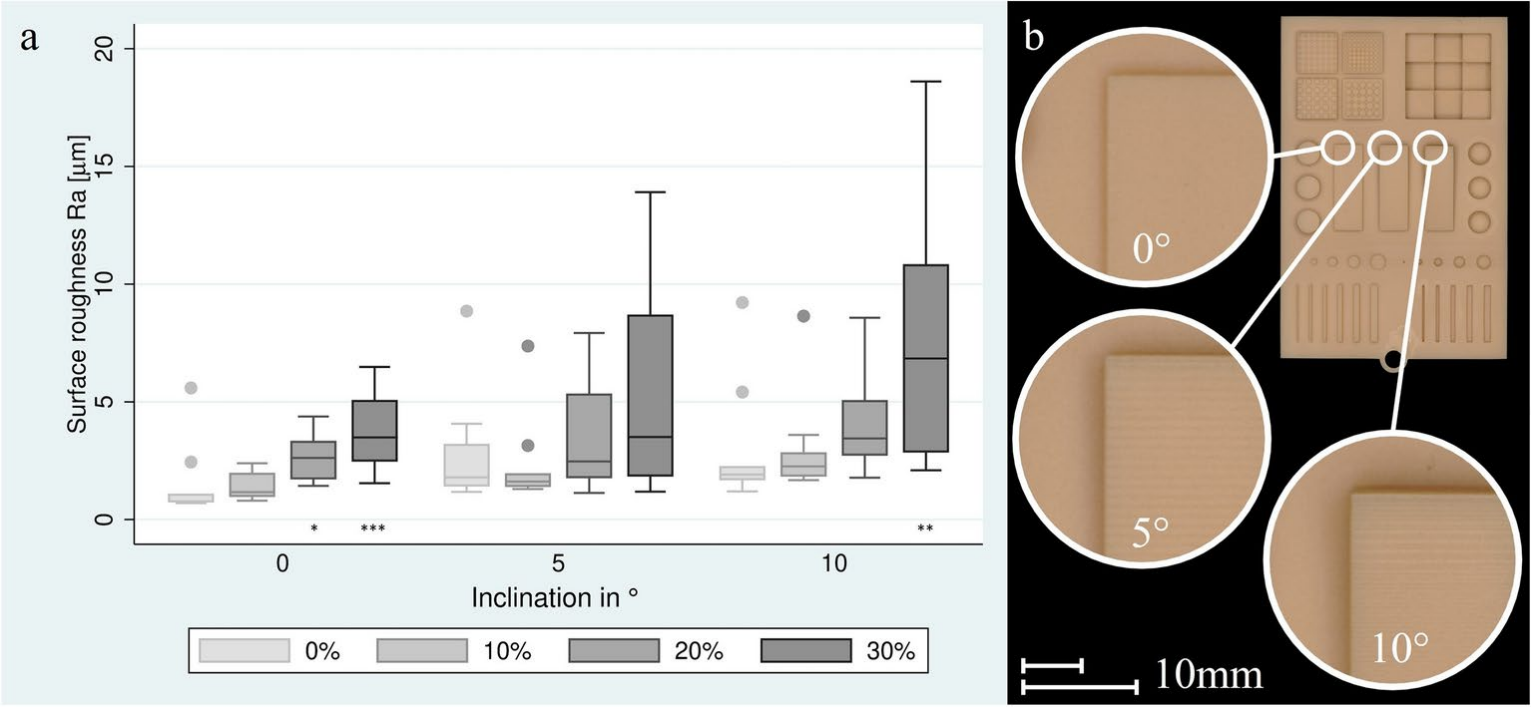
(**a**) Surface roughness on planes with increasing inclination; (**b**) Overview on test structure with visible “stair-stepping” when washing with 99.9% IPA. Significance against post-rinsing with non-contaminated IPA: *p < 0.05, **p < 0.01, ***p < 0.001.

The change in surface roughness on planes with increasing inclination is shown in Fig. 5a and Table 2. The staircase effect of the surfaces with the inclination of 5° and 10° is clearly visible on the specimen cleaned with IPA with a contamination of 0 wt% (Fig. 5b). Significant deviations were found in the 30 wt% contamination group at 0° and 10°, and in the 20 wt% contamination group at 0°.

**Table 2:** Surface roughness Sa [in µm] on planes with increasing inclination after post-rinsing.

	Inclination of the planes	0°	5°	10°
Ramp Structure	0 wt% Contamination	1.54 ± 1.43	2.71 ± 2.22	2.89 ± 2.39
	10 wt% Contamination	1.43 ± 0.55	2.31 ± 1.76	2.96 ± 1.98
	20 wt% Contamination	2.57 ± 0.90*	3.37 ± 2.15	4.07 ± 2.05
	30 wt% Contamination	3.67 ± 1.55***	5.11 ± 4.09	7.95 ± 5.47**

Significance against post-rinsing with non-contaminated IPA: *p < 0.05, **p < 0.01, ***p < 0.001.

## Discussion

### Main findings

In this study, the influence of monomer/resin contamination in the isopropanol solution for post-rinsing on the accuracy of test specimens was investigated. Therefore, test specimens were additively manufactured and post-processed. In the post-rinsing step, different degrees of monomer/resin contamination (0 wt%, 10 wt%, 20 wt% and 30 wt%) were added to simulate progressive resin accumulation, as it occurs when the IPA is not replaced. The samples were then scanned and superimposed with the original sample design. By comparison, deviations were recorded as mean and maximum deviation and root mean square in profile and 3D-view. Furthermore, the surface roughness was also measured. Significant deviations are listed in Table 3 and are used to determine the main findings.

**Table 3:** Overview of statistically significant deviations from CAD-file.

	Test geometry		Contamination			Main findings
			10 wt%	20 wt%	30 wt%	
Set of fine features	Area 1	Max. deviation	-	-	+	Significant deviations mostly occurred from a contamination of 30 wt%@@Small gaps (250 µm) are more difficult to flush, thus increasing resin accumulation
		RMS	-	+	+	
	Area 2	Max. deviation	-	-	+	
		RMS	-	-	+	
	Area 3	Max. deviation	-	-	+	
		RMS	-	-	-	
	Area 4	Max. deviation	-	-	+	
		RMS	-	-	+	
Small Pin Structure	500 µm	Av. deviation	-	-	+	Few significant deviations with contamination of 30 wt%@@Deviations have occurred in the range of small “outside” structures ≤1000 µm
		Max. deviation	-	-	+	
	1000 µm	Av. deviation	-	-	+	
		Max. deviation	-	-	-	
	1500 µm	Av. deviation	-	-	-	
		Max. deviation	-	-	-	
	2000 µm	Av. deviation	-	-	-	
		Max. deviation	-	-	-	
	2500 µm	Av. deviation	-	-	-	
		Max. deviation	-	-	-	
Small Hole Structure	500 µm	Av. deviation	-	+	-	Hardly significant deviations with contamination of 30 wt%@@Deviations have occurred in the range of small “inside” structures ≤500 µm
		Max. deviation	-	-	+	
	1000 µm	Av. deviation	-	-	-	
		Max. deviation	-	-	-	
	1500 µm	Av. deviation	-	-	-	
		Max. deviation	-	-	-	
	2000 µm	Av. deviation	-	-	-	
		Max. deviation	-	-	-	
	2500 µm	Av. deviation	-	-	-	
		Max. deviation	-	-	-	
Ramp Structure	0°		-	+	+	Significant deviations mostly occurred from a contamination of 30 wt%@@When post-rinsing with IPA contaminated with resin, it can be assumed that the steps are filled with excess resin
	5°		-	-	-	
	10°		-	-	+	

Statistically significant deviations are marked with (+), non-significant deviations with (-). The results of 0 wt% contamination group were taken as reference.

While our study specifically investigates Model V2 resin, the presence of similar monomers in different resin formulations and therefore similar dissolution behaviour with IPA, may lead to comparable effects. A high degree of comparability can be assumed for resins, whose main components are also methacrylate monomers and urethane dimethacrylate. This contentration decreases with further distance from the surface (e.g., bisphenol A dimethacrylate or other methacrylate ester monomers, or those containing ceramic particles) may not follow the same trend and need to be investigated in further studies.

The following findings were obtained for all test structures and measurement parameters. No statistically significant deviations were observed up to a contamination level of 10 wt%. However, these also occurred only sporadically in the 20 wt% contamination range, which is a realistic concentration if, for example, no replacement is provided for economic reasons or due to negligence. Only at contamination levels of 30 wt% the most notable deviations occurred. Moreover, the scattering of the results was greatest in this instance and therefore the most difficult to predict. In addition, small structures proved to be more problematic than large and inverse structures more problematic than “outside structures”, which is due to the small inlet opening and the lack of IPA flow around those. In contrast to the manufacturer’s specifications, post-rinsing of components with large, smooth surfaces without small, inverse structures therefore appears to be quite possible with a resin contamination of up to 20 wt%, whereas significant deformations are to be expected with a higher contamination.

However, all samples, even those treated with pure IPA, showed, although not necessarily statistically significant, deviations from the sample design as shown by previous studies and dicripted later. In addition, some structures could not be depicted due to technical specifications e.g. the resolution of the printing system used (small hole, diameter 500 µm).

Regarding the “set of fine features” structure, RMS showed significant deviations mainly at a contamination level of 30 wt%. A contamination of 20 wt% also led to significant deviations in the test structure at the largest geometries and the widest gaps. The maximum deviations resulted in significant deviations in the 30 wt% contamination group.

In the case of small pins and holes, significant mean and maximum deviations mainly occurred at a contamination level of 30 wt%. For the small pins and holes, only structures < 1500 µm were affected by significant deviations. For structures > 1500 µm, no significant deviations occurred in most cases.

At a plane inclination of 5°, no significant deviations in surface roughness were found. This suggests that the steps formed by the different print layers were added and masked by excess resin. At planes with an inclination of 0°, significant deviations took place from 20 wt% contamination onwards. The surface here is formed from a single layer of print and no masking can take place. Due to the plane inclination of 10° and the resulting height of the structure, the surface was formed from the largest number of layers in this test setup. In this case, the steps of print layers obviously could not be masked by excess resin. Significant deviations occurred here.

For all geometries the contamination gradient and the local contamination difference can influence the effectiveness of the removal of the resin. If resin dissolves from the surface of the sample into IPA, there is a local increase in the monomer concentration at the interface between solvent and sample. This contentration decreases with further distance from the surface. This phenomenon is also influenced by the circulation of the solvent. Inside structures are less well circulated by IPA than outside structures. Due to the lack of circulation, it is conceivable that the local contamination in the vicinity of inside structures is greatly increased compared to the average contamination of the solvent as a whole. Consequently, a greater amount of resin adhering at the surface is prevented from being dissolved. In contrast, exterior structures benefit from enhanced circulation, resulting in a substantially smaller local increase in contamination and facilitating effective dissolution of resin at the surface.

Furthermore, inner edges offer a more retentive surface for cohesion and adhesion effects in comparison to smooth surfaces or outer edges. Consequently, inner edges are more influenced by resin adhesion. The width, depth, number, and absence of retention niches have a significant impact on adhesion. For instance, adhesion to the intricate structure of the "set of fine features" was more strongly affected by adhesion than the large, smooth surfaces of the inclined planes. Furthermore, it is plausible that, particularly in instances of high IPA purity, there may be additional dissolution or leaching of unpolymerized residual resin from the uncured sample. This residual resin, if they had not been dissolved beforehand, would have been post-polymerized during the curing process.In some cases, a material deficit was observed in the area of the outer edges in the cured samples, which could be attributed to this phenomenon.

#### Evaluation of the results in context of the current state of research

The study examined the effect of post-rinsing on surface deviations, specifically root mean square (RMS), using a test geometry consisting of a set of fine features. The measured values of RMS and maximum deviation increased with higher contamination and reduction of poorly flushable interstitial spaces. The average RMS for washing with 99.9% IPA ranged from 29 ± 4 µm to 51 ± 4 µm, and it increased up to 89 ± 44 µm with higher contamination. These results fall within the range of previous studies that also investigated surface deviations using RMS [16, 18, 39] and focused on the type and duration of post-rinsing, rather than the type or degree of solvent contamination and used therefore all uncontaminated, pure 99.9% IPA. The achieved RMS values in automated post-processing ranged from 20 ± 1 µm to 126 ± 15 µm [16], with values of 34 ± 4 µm and 56 ± 7 µm reported by Alharby et al. and Osman et al. falling in between our results [17, 39]. It is worth noting that the maximum deviation of a three-dimensional surface was also recorded, as a single larger deviation can negatively affect the fit. After post-rinsing with 99.9% IPA, the results ranged from 124 ± 42 µm to 209 ± 17 µm on average, and increased to 299 ± 65 µm with increasing contamination. Variations in results were observed even when washing with the same contamination, highlighting the influence of the AM component, as wider gaps were rinsed more effectively with less resin adhesion. Even with minimal contamination, deviations from the computer-aided design (CAD-) file were observed.

Another parameter investigated in the study was the mean deviation of a two-dimensional cross-section using small pin and hole structures. The absolute values were normalized to the width of each structure. The average absolute values ranged from 139 ± 43 µm to 203 ± 54 µm for outside structures, and from 93 ± 57 µm to 578 ± 83 µm for inside structures, with an increase in values observed with increasing contamination. Some values in this study substantially differed from previous studies due to the smaller and more detailed measurement structure capturing smaller inaccuracies. Previous studies [4, 30, 33] focused mainly on test specimens with large, smooth surfaces with measured values ranging from 68 ± 1 µm [30] to 118 ± 22 µm [33].

In the case of additive manufacturing of irregular shapes with inclined surfaces, step formations can be observed, with more pronounced step formations at lower z-resolution (greater layer thickness). Post-rinsing with resin-contaminated IPA is expected to fill the steps with excess resin, making them less visible and resulting in a more organic shape and relatively reduced surface roughness (Sa). In fact, the ramp of the 5° slope did not show any significant deviations, which suggests that the printed steps were masked by the excess resin. At an inclination of 0°, the surface is formed from a single printed layer. Here, resin accumulation led to significant deviations. At an inclination of 10°, the largest number of layers in this setup was cut at the steepest angle, and the steps could not be masked and significant deviations occurred. The assumption that steps are masked by excess resin and lead to lower surface roughness must therefore be partially rejected. In previous studies, surfaces with inclinations of 0°, 45°, and 90° exhibited surface roughness values of 0.39 ± 0.10 µm, 1.09 ± 0.07 µm, and 0.45 ± 0.23 µm, respectively [5]. This indicates that the surface roughness increased when multiple layers were cut at an angle and decreased when cut vertically. Similarly, Mayer et al. [8] reported surface roughness values ranging from 0.86 ± 0.32 µm to 3.38 ± 1.21 µm for horizontal layers of different resins after AM, post-rinsing, and post-curing, which aligned with the trend observed in this study for flat and angled surfaces.

## Conclusions

Washing processes of AM produced test specimens with pure IPA or with a maximum monomer contamination of up to 10 wt% resulted in consistently small deviations of the parameters examined (mean, maximum, RMS-deviation, surface roughness). Models with sharp-edged inverted and raised structures, fine model details but also smooth surfaces could be reliably imaged while maintaining this contamination limit. However, different to the manufacturer’s specifications, a monomer contamination of up to 20 wt% did not necessarily had a significant influence on the model accuracy. Consequently, workpieces with larger smooth surfaces or anatomical shapes without sharp edges or small, inverse structures could be produced after a washing process without major inaccuracies. In contrast to the manufacturer’s instructions, replacing the IPA is not absolutely necessary here. Contamination levels of 30 wt% had a significant impact in most cases, highlighting the need to replace the IPA in this scenario. As a result, the null hypothesis H_0_ stated at the beginning [increasing monomer contamination of the isopropanol solution has no significant effect on the accuracy (mean, maximum, RMS-deviation, surface roughness) of components that have completed post-processing] has to be (partially) rejected: an increasing monomer concentration has an influence on the accuracy, but in most cases only from a contamination of > 20 wt%.

## Supplementary Information

Supplementary Information 1.

## Data Availability

The datasets used and/or analyzed during the current study are available from the corresponding author on reasonable request.
